# Why the Delay? Factors Predicting Step 1 Delay

**DOI:** 10.1007/s40670-025-02349-0

**Published:** 2025-03-10

**Authors:** Jeannette Manger, Lindsay Benedik, Michael Matott, Chasity B. O’Malley

**Affiliations:** 1 Department of Medical Education, Boonshoft School of Medicine, Wright State University, 3640 Colonel Glenn Hwy, Dayton, OH 45435 USA; 2 Department of Pharmacology, Physiology, and Neuroscience, The New Jersey School of Medicine, Rutgers University – Newark, Newark, NJ USA

**Keywords:** Step 1, Active learning, Student performance, Medical education

## Abstract

Few studies have examined Step 1 delay trends in active learning or identified other factors that could influence Step 1 delay, which could inform interventions for struggling students. Our study examines factors across an active learning curriculum that could potentially predict delaying Step 1. Using a multiple logistics regression model, we found that performance on NBME final exams in our major systems-based modules later in the curriculum correlated with the decision to delay, but that formative assessments were the earliest strong predictors. These findings indicate that successful participation in an active learning curriculum positions students for success in Step 1.

## Background

Step 1 readiness remains a stressful subject for medical students, as they are only afforded limited attempts, and delay or failure can lead to significant delays in their future education and have serious professional consequences. A challenge in the pre-clerkship medical school curriculum is finding predictive factors to allow for early intervention and support for students who are struggling. In this study, we examine predictive factors within our exclusively active-learning curriculum that could be used to predict Step 1 delay.

Our pre-clerkship curriculum, referred to as Foundations, is 41 weeks in duration and consists of basic sciences integrated with clinical sciences including Origins (Biochemistry, Cell Biology, Genetics, Neoplasia), Human Architecture I and II (Anatomy), Host and Defense (Immunology and Microbes), Staying Alive (Cardiovascular, Pulmonary, Renal), Beginning to End (Endocrine, Reproductive, Gastrointestinal), and Balance, Control, and Repair (Neuroscience, Psychiatry, Dermatology, Musculoskeletal) (Table 1) [1]. Students also engage in longitudinal courses such as Clinical Medicine (clinical skills focused) which occurs throughout years 1 and 2, as well as Scholarship in Medicine (research focused) and Biostatistics, which occur in year 1. Our curriculum challenges students to build knowledge through self-directed learning and assess their understanding through Peer Instruction, Team-Based Learning, and Problem-Based Learning [2–4]. This approach relies entirely on active learning approaches previously shown to support basic science reasoning in undergraduate medical students [5–8]. Because evidence indicates that success on National Board of Medical Examiners (NBME) exams and questions correlates with future success, our assessments include many of these elements [9–11]. Students receive formative feedback regularly throughout a course via multiple choice question (MCQ) exams written in NBME format, while summative feedback is provided through NBME customized assessments at the end of the course [12, 13].

**Table 1:** Foundations of clinical practice curriculum timeline overview

Year 1	Clinical Medicine I	Origins	Human Architecture I	Origins (cont’d)	Host and Defense	Staying Alive
	*2 weeks*	*5 weeks*	*5 weeks*	*9 weeks*	*6 weeks*	*12 weeks*
Year 2	Human Architecture II	Beginning to End	Balance, Control, and Repair	Dedicated Step 1 Study/Take Step	Clinical Medicine Doctoring	Clerkships
	*4 weeks*	*12 weeks*	*10 weeks*	*6 weeks *	*4 weeks *	*Continue to years 3 and 4*

Table 1 shows the major modules of the foundations of clinical practice

While other studies have shown that some tests such as the Comprehensive Basic Sciences Self-Assessment (CBSSA) are correlative of Step 1 scores immediately prior to taking the exam, our goal was to find earlier predictors of Step 1 delay and success to allow for earlier intervention [10, 16]. In some cases, academic performance in the medical school curriculum has been shown to positively predict Step 1 performance, but many of these studies were completed in traditional lecture environments [15, 17–21]. We not only wanted to show predictive value in an active learning environment, but also to earlier identify struggling students and provide meaningful interventions. While the consequences of Step 1 failure are clear, a delay in our curriculum puts students at a disadvantage [12, 21]. Our students are permitted to begin clerkships if they have achieved a passing Step 1 score or have taken the Step 1 exam prior to the first day of clerkship block 1; anything later than this could reduce the available time for Step 2 prep, elective selection, completion of graduation requirements, and ultimately graduation. Because our curriculum currently has minimal guidelines on milestones for taking Step 1 on time, understanding predictive factors could inform these decisions, as well as provide opportunities for earlier interventions. Here, we aim to show factors, such as formative and summative assessments, that contribute to Step 1 delay in an active learning curriculum.

## Activity

All students enrolled in BSOM starting with the class of 2021 to the class of 2025 were considered for inclusion as the class of 2021 was the first cohort in the active learning Wright Curriculum. Students were excluded if they failed a module or took a leave of absence for any reason which subsequently resulted in changing cohorts. Students included in the analysis were first assigned to an appropriate cohort based upon the anticipated graduating class. They were then divided into two groups: those who delayed taking Step 1 defined per our curriculum committee as those that did not test prior to the start of clerkship block 1 and those who did not delay Step 1. Variables for admission and the Wright Curriculum which included MCAT scores, average individual MCQ (multiple choice question exam) scores, and NBME customized assessment scores were selected as potential predictors for Step 1 delay. Due to the consistent use of the NBME final examination, only four modules (Origins, Staying Alive, Beginning to End, and Balance, Control, Repair) in the Foundations portion of the Wright Curriculum were selected for analysis. Testing of data distribution showed a normal distribution; therefore, parametric statistics were used. Prior to combining all cohorts into a single data set for further analysis, a two-way ANOVA was performed on all variables using cohort year as the second independent variable to ensure cohort year did significantly impact results. A one-way ANOVA was used for analysis of MCAT scores and number of delayed students across cohorts to reduce the risk of a type I error in comparing across multiple groups. Unpaired *t*-tests between delayed and non-delayed students were completed for all other individual continuous variables after being combined into a single pooled data set. The multiple logistic regression model was then used to determine the predictive value of each variable with a delay in taking Step 1.

Demographic data was provided by the Office of Admissions and is provided in aggregate for the class cohorts as a whole. Race and ethnicity data are optional and therefore several students have opted to exclude this information from their file. Percentages provided for race were based upon the number of students providing an answer compared to the cohort total.

Data were collected and processed in Excel. Statistical tests were performed using SPSS (version 29, IBM) and figures were made using GraphPad Prism (version 10.02).

This study was conducted under the Institutional Review Board protocol approval #6553 from Wright State University IRB.

## Results and Discussion

The demographic makeup of the cohorts primarily consisted of White, non-Hispanic individuals, aged 23–24 years of age, and identified as female (Table 2).

**Table 2:** Demographic makeup of the cohorts

Cohort (class of)	% White, non-Hispanic	% male	Mean age ± std dev (years)
2021 (*n* = 105)	71.43	46.67	23.6 ± 3.3
2022 (*n* = 117)	60.68	43.59	24.1 ± 2.6
2023 (*n* = 111)	44.14	36.94	24.2 ± 3.1
2024 (*n* = 113)	53.98	40.71	24.6 ± 3.4
2025 (*n* = 123)	60.16	45.53	24.3 ± 3.4

**Fig. 1:**
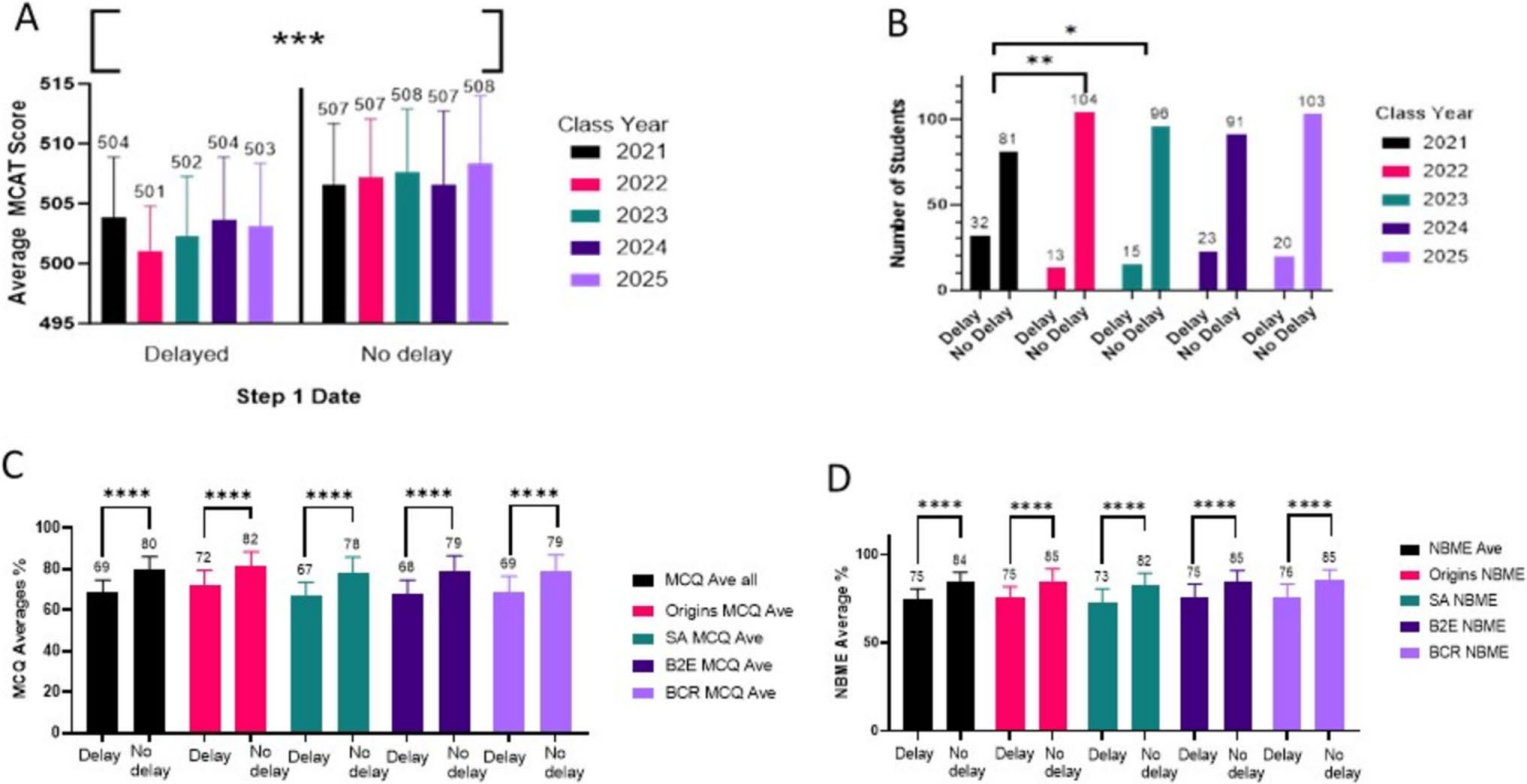
Step 1 delay factors. **A** Step 1 delayers had a significantly lower MCAT score entering the medical school (one-way ANOVA, *p* < 0.0001). **B** Number of students who delayed taking Step 1 compared to those that did not delay. The class of 2021 had significantly more delayed students than the class of 2022 (*p* = 0.0056) and class of 2023 (*p* = 0.0247) (one-way ANOVA, Tukey’s multiple comparison). **C** Delayed students had significantly lower MCQ averages compared to non-delayed students for the four major modules of the Foundations curriculum (unpaired *t*-test, *p* < 0.0001). **D** Delayed students had significantly lower NBME final grades compared to non-delayed students (unpaired *t*-test, *p* < 0.0001). Acronyms: MCAT, Medical College Admission Test; MCQ, Multiple Choice Question exam; SA, Staying Alive module; B2E, Beginning to End module; BCR, Balance, Control, Repair module

The average MCAT scores for non-delayed vs delayed students were significantly different in all cohorts (class of 2021 506.5 ± 1.1 vs 503.8 ± 1.1, *p* = 0.0152; class of 2022 507.2 ± 1.4 vs 501.1 ± 1.4, *p* < 0.0001; class of 2023 507.6 ± 1.5 vs 502.3 ± 1.5, *p* = 0.0003; class of 2024 506.6 ± 1.4 vs 503.7 ± 1.4, *p* = 0.0373; class of 2025 508.3 ± 1.4 vs 503.2 ± 1.4, *p* = 0.003) (Fig. 1A).

The class of 2021 had significantly more students delay (*n* = 32 delayed; 81 non-delayed) than the class of 2022 (*n* = 13 delayed; 104 non-delayed; *p* = 0.0056) or the class of 2023 (*n* = 15 delayed; 96 non-delayed; *p* = 0.0247) (Fig. 1B). There was no significant difference between any of the other cohort comparisons. This significant delay in 2021 was likely multifactorial including factors related to the COVID-19 pandemic, students delaying in an effort to increase their score, and lack of policies regarding timing of Step 1.

Students who delayed Step 1 had lower average MCQ exam scores in all modules included in our analysis (Fig. 1C). For all modules, students that delayed Step 1 (*n* = 103) scored lower than the non-delay students (*n* = 474) with most modules showing a difference of more than 10 percentage points (MCQ overall average 69.01 ± 0.77 vs 79.74 ± 0.77, *p* < 0.0001; Origins 72.25 ± 0.75 vs 81.67 ± 0.75, *p* < 0.0001; Staying Alive 66.86 ± 0.80 vs 78.36 ± 0.80, *p* < 0.0001; Beginning to End 67.84 ± 0.77 vs 79.47 ± 0.77, *p* < 0.0001; Balance, Control, Repair 69.02 ± 0.82 vs 79.37 ± 0.82, *p* < 0.0001) (Fig. 1C).

Students who delayed Step 1 scored lower on the end of module NBME exams in for all modules (NBME overall average 74.99 ± 0.60 vs 84.41 ± 0.60, *p* < 0.0001; Origins 75.28 ± 0.74 vs 85.00 ± 0.74, *p* < 0.0001; Staying Alive 82.42 ± 0.77 vs 73.02 ± 0.77, *p* < 0.0001; Beginning to End 75.37 ± 0.72 vs 84.65 ± 0.72, *p* < 0.0001; Balance, Control, Repair 76.17 ± 0.66 vs 85.46 ± 0.66, *p* < 0.0001) (Fig. 1D).

To examine whether performance in the Foundations curriculum could be a predictor of a student delaying Step 1, we used a multiple logistic regression on the pooled data set and determined that performance on MCQ exams for Staying Alive and the NBME exam for Origins had some predictive value (*Z* = 2.563, *p* = 0.0104 for Staying Alive MCQ average and *p* = 0.0248 for Origins NBME) (Table 2). Performance on the Beginning to End MCQ exams had slightly more predictive value (*Z* = 2.603, *p* = 0.0092) (Table 3).

**Table 3:** Variables which were predictive of whether a student would take their Step 1 exam on time. * indicates *p* < 0.05; ** indicates *p* < 0.01; *** indicates *p* < 0.001. Acronyms: *MCAT* Medical College Admission Test, *MCQ* Multiple Choice Question exam, *SA* Staying Alive module, *B2E* Beginning to End module, *BCR* Balance, Control, Repair module

Variable	|*Z*|	*P* value	*P* value summary
Intercept	3.528	0.0004	***
MCQ Ave Origins	0.8520	0.3942	ns
MCQ Ave SA	2.563	0.0104	*
MCQ Ave B2E	2.603	0.0092	**
MCQ Ave BCR	1.167	0.2432	ns
NBME Origins	2.245	0.0248	*
NBME SA	1.273	0.2032	ns
NBME B2E	0.1102	0.9122	ns
NBME BCR	3.373	0.0007	***
MCAT	1.970	0.0488	*

Various factors contribute to the need to delay Step 1. Our study found several curricular indicators which may assist in the identification of students that are at risk for Step 1 delay to facilitate early intervention. Despite the number of delayed students, those that delayed Step 1 had a pass rate of 83.49% compared to 97.26% pass rate for those that did not delay which may more broadly indicated the value of early assessments on not only delay, but first time pass rate on Step 1. As with previous literature, MCAT scores correlated with delaying Step 1; however, performance within the curriculum was the greatest predictor of Step 1 delay [22–24]. This suggests that participation in an active learning curriculum can facilitate Step 1 success in students with lower-than-average MCAT scores. Overall, the in-module MCQ performance in core system-based modules was a better predictor of Step 1 delay than the end of module NBME examinations, with the exception of the NBME exam penultimate to taking Step 1. Several factors likely contributed to this finding; in-module MCQs are purposefully designed to be more challenging and test a more in depth understanding of concepts, while the NBME final examinations were designed to evaluate the summative understanding of module content. Additionally, the MCQs are considered lower stakes, as the cumulative performance on these exams in addition to engaged learning determine the eligibility to take the NBME final exams which are the final determinants of whether a student passes a module or not. While the NBME final examination for Balance, Control, Repair had a high predictive value, it would not be an effective “early predictor” as it is immediately followed by dedicated Step 1 study time. Unsurprisingly, the modules with the highest percentage of Step 1 content were the most highly predictive of Step 1 delay: the Staying Alive module consists of approximately 18–26% of the content reflected on Step 1 and Beginning to End reflects approximately 18–26% of the content [25].

## Conclusion

Despite the transition to Pass/Fail, Step 1 remains a stressful checkpoint for medical students [26, 27] Using data from our active learning curriculum, we found several factors which may be predictive for a student that may be at risk of delaying Step 1, one of which can be determined at the end of the first year. We found no evidence of a significant change in students delaying Step 1 across our timepoints; our future goal is to provide earlier interventions for at risk students and better counsel students who could benefit from a necessary delay. An important next step at our institution is to generate resources for students at risk of delaying Step 1 and explore the value and impact of these interventions. Because the most predictive first-year course occurs at the end of the academic year, a practical approach would be to provide these interventions during the summer and throughout the second year. Our current study not only shares the usefulness of formative assessments as potential predictors of Step 1 delay, but it also provides a novel context for such studies: the active learning curriculum. We encourage other institutions to explore their own unique formative assessment landscape to best support their students.
